# Imagination in Medicine

**Published:** 1895-10

**Authors:** Stuart Close

**Affiliations:** Brooklyn, N. Y.


### IMAGINATION IN MEDICINE.

STUART CLOSE, M. D., BROOKLYN, N. Y.

Certain important elements of success in healing the sick have been very largely, if not entirely, overlooked. These elements are not apparent on a superficial examination of the subject. In their secondary relations they have been recognized, and successful physicians employ them, more or less unconsciously. Success would be greatly enhanced if they were recognized primarily, and the faculties and qualities referred to were intelligently developed and cultivated.

Reference is made principally to that faculty of the human soul we call Imagination—the least understood and the most neglected faculty we possess, while at the same time it is one of the most important. Its right and scientific use is the key to success. Its misuse or neglect leads to a train of evils which can only be hinted at here.

Other professions than the medical recognize and cultivate the imagination with reference to the needs of their special fields, although the world at large thinks of it as the almost exclusive possession of artists, poets, and dreamers, and values it in proportion as it appreciates the product of their work.

It could very easily be shown that many of the grandest accomplishments which mark the progress of the world have had their origin in the exercise of this faculty, but the object of this paper is to show some of its relations to the art and science of homœopathic medicine. We shall find the basis of our position in Hahnemann's philosophy of life and diseases. In the Hahnemannian philosophy, man is regarded as a triune being, composed of a material body, animated and controlled by an immaterial vital principle for the use of the indwelling rational spirit (*Organon*, § 9). What is meant by the material body is perfectly clear. His doctrine of the second element is not quite so clear, but when the expressions used in the various sections of *The Organon* are compared, it seems probable that he held substantially what might be called the mystical theory of the soul, as promulgated by the great Mystics of the ages from Socrates and Plato down, including Bœhme, Paracelsus, and Swedenborg. Hahnemann does not devote much time to speculation, but confines his attention principally to the development of the doctrine of the "vital force," or vital principle, also called "dynamics," and "spirit-like autocracy," which is that aspect or condition of the soul concerned in what we call disease.

He shows that disease is a derangement of this vital force—of the harmonious relation between the soul and the body, in which that spirit-like autocracy is partially lost, and the tendency is toward further loss, and final complete separation between soul and body, or what is called death.

The vital force, therefore, is that principle which maintains the harmonious relation between the soul and the body—or Life. The soul is the real man, intangible but substantial, invisible under ordinary circumstances to the physical eye, but the counterpart, organically, of the material body, which it animates and controls.

The body is the instrument and means by which the soul is brought into objective existence, and into relations with the eternal world. The "indwelling rational spirit" is the divine part—the direct offspring of God—immortal, supreme; conscious in all men, manifesting itself through the voice of Conscience, but active in proportion to the degree of spiritual development of the individual.

Man, therefore, in the Hahnemannian philosophy, is essentially a spiritual being. This opens a way for the consideration of the mental, psychical, and spiritual relations of disease.

It may be worth while in passing to remark that the therapeutic system of Hahnemann is the only one based upon this threefold nature of man, and the only one having a principle of treatment applicable alike in the three spheres.

We may confidently claim for the principle, universality of application. Wherever derangement exists and remedial measures are to be applied, they must be in accordance with the homœopathic principle. Man, the microcosm, is only a miniature of the universe—the macrocosm. The same laws hold in both. The world will learn this by and by. Great will be the changes wrought in the constitution of things when it does.

Having clearly defined man's true nature and constitution, Hahnemann was ready to study the influences which affect him for good or evil. His philosophy opened his eyes to the influence of those finer and more occult forces which affect man, and enabled him to include their results in the scope of his curative process. No one before his time had been able to "minister to a mind diseased." Under his method the mental states and symptoms are advanced to a position of the first importance as indications for treatment. The mental includes the emotional and psychical, and so the whole man is brought under the beneficent dominion of homœopathic law.

The effect of the exercise of the emotions has been referred to as among the prominent causes of disease. Anger, anxiety, fright, grief, indignation, jealousy, unhappy love, mortification, even excessive joy, are all depressing and disturbing influences, and produce characteristic sick conditions, which we must treat and cure. Originating in the psychic sphere, through the disturbance of that principle which maintains the harmonious relation betwixt soul and body, their effects are very soon manifested in the material body, in some organic change. I have in more than one case traced cancer directly to long-continued anxiety and grief, for example.

But all these emotions, as well as their opposites, have their foundation and source in that wonderful faculty of the soul we call Imagination. It is the soil from which they spring and grow—the sphere in which they "live and move and have their being." Imagination first pictures the ground or condition, and then the consequence. The thought, feeling, or emotion follows.

All recognize in a general way the effect, good or evil, of what is called one "personality" upon another, especially where the sick are concerned. Who does not know the almost irreparable harm done by some sympathizing sister who comes into the room on tip-toe, and with every show of affection and sympathy proceeds to inform the patient, in sepulchral whispers, how sick she looks, how she must suffer, how serious the doctor and the friends look, and then launches out upon a circumstantial description of the sickness, death, funeral, and burial of some one who was afflicted with a similar disease. She goes and leaves the shadow of death behind her. She has drawn a picture from her own morbid imagination and impressed it upon that of her patient, made abnormally sensitive already by sickness. Fear and dreadful forebodings depress the vitality of the patient, perchance beyond recovery.

Who also does not recognize the blessing brought to the sick one by the quiet, cheery, bright, hopeful soul who comes into the sick-room like a ray of sunshine, bringing a flower, a bit of pleasant news, and an encouraging word.

The physician recognizes these visitors, condemning savagely the one, forbidding her the house, while welcoming and indorsing the other. It is doubtful whether all physicians take the lesson home to themselves, however, as clearly as they might.

One, partly grasping the idea, assumes an exaggerated gayety, enters the sick-room boisterously, greets the patient in a tone of voice which makes the windows rattle, chaffs and banters him, and assures him he will be all right in a day or two, while he is engaged in putting up the medicine, to the selection of which he has probably given hardly more than a passing thought. The patient is not deceived by such assumption, nor benefited by such visits.

Another goes to the other extreme. Clothed in black, he enters the room with solemn visage, views the patient and his surroundings, feels his pulse, looks at his tongue, asks some questions, shakes his head ominously, or frowns over some of the replies, gives his directions, and departs expressing a hope that the patient will be better to-morrow with an air which implies that he fears it will not be so. It is probable that his intentions were good. He meant to look wise, dignified, self-possessed. He succeeded only in looking funereal.

In both these cases the imagination is at fault. A false and distorted ideal has been pictured. The profession is favored occasionally with advice on these matters—the necessity for cheerfulness and a confident air in the sick-room—but little or no light is thrown on the method of attaining or developing these desirable qualities. Those who offer this advice usually speak in generalities, and treat the matter as if these qualities could be assumed at will. They do not realize that assumed qualities are worthless, and deceive only for a short time at best. Neither do they realize the source of the genuine qualities or how to attain them, though they recognize the quality when it comes to them unconsciously. Hahnemann gives the key to solution of the problem in Section 3: "When the physician clearly perceives *what is to be cured* * * *." This refers back to his philosophy of the nature and constitution of man. The corollary of this statement is that it is quite as important to see what is *not* to be cured. In other words, the physician is to form, by the exercise of his imagination, an ideal of his patient *in health.* This corresponds to the real man, the spiritual man, who *is not sick* or in need of treatment. Let him vividly picture his patient in all the strength and beauty of perfect health, with every function harmonious. Let him not permit his mind to dwell for an instant upon any derangement which exists until this image has been formed. When it has taken full possession of the mind it becomes a standard of comparison and a basis from which to work in making his examination. Using all his anatomical and physiological knowledge to assist him in keeping this standard in mind, let him proceed now to note every deviation from the standard in function and sensation. Mark the attitude of mind. He is to gather the symptoms for the purpose of comparison—first, to ascertain *what in the case is to be cured*, and second, with the symptoms of proved drugs in order to find the similar remedy. He *is not* to consider the symptoms for the purpose of erecting another imaginary structure to be called "The Disease," which it is his duty to destroy.

Disease is not an entity, and is not even to be imagined as such. This maxim has become almost hackneyed from frequent repetition by all our best teachers. Yet it does not seem to be fully understood, and the need for its repetition appears to grow more imperative than less. Perhaps it is because there is no firm and intelligent conviction of its essential truth, and a failure to trace clearly the relation between cause and effect.

Once let the idea of disease as an entity take possession of the mind of the physician, and dwell in his imagination as such, and not only will he fail to produce a correct mental and psychical impression upon his patient, but he will frequently fail to find the similar remedy. Remedies are not similar to disease, but to groups of symptoms, which frequently have no resemblance to any disease.

The imagination of the up-to-date doctor seems to be occupied very largely with visions of microbes. The microbe is rapidly growing more numerous and more ferocious, and his ravages more appalling, as attention is directed to him and his habits and appearance are studied. He seems to rejoice in the furore he has created and thrive in the pabulum in which he is cultivated. He is the Homunculus of Faust—the veritable Frankenstein of modern science, who promises to destroy his creator, while he is pluming himself for flights into realms hitherto uninvaded. This is the modern way of regarding disease as an entity. Its results in practice are plainly seen. The theory has taken firm hold upon the mind and imagination of a large part of the profession, and the lay world suffers the consequences. In the attempt to found a mode of treatment upon this theory more *homicides* than *germicides* have been discovered. The physician may sometimes wonder why, in spite of carefully selected remedies, his patient does not seem to improve. May not the explanation sometimes be found in this wrong attitude of the mind and imagination of the physician toward the patient? With his mind occupied by an image of disease which he has conjured up, named, and clothed in all its pathological terrors, an appearance of cheerfulness and hope will be all he is able to accomplish. He is himself, half unconsciously, a prey to fear, and this influence is communicated to his patient in all its depressing effect. There is a subjective and subconscious mind as well as an objective and conscious one in man. The subjective mind is automatic and absolutely sleepless. Within it occur the phenomena of dreams, of mental impressions, all the phenomena of telepathy or thought-transference, and of hypnotic suggestion. It is frequently most active in sickness, and the patient is then most sensitive to impressions and suggestions, or what is called unconscious influence. The subjective mind of the patient easily penetrates the physician's assumption of cheerfulness, and reads what is going on in the precincts of his subconscious mind. Finding there phantoms and forebodings, doubts and hesitation, it is shocked, depressed, injured.

If the physician is sensitive, he will himself be depressed in vitality, perhaps made seriously ill. A lady remarked to me once that it appeared to her if there was anything in "mental impression," physicians would be sick all the time, as they were so constantly studying and thinking of disease. The true physician is not, and, if he is to be successful as a healer, *must* not be constantly thinking of disease. He is to be thinking of *health.* His attention and desire are both to be turned toward an ideal of health—not disease. In so doing he not only preserves himself, but helps his patient, through the medium of unconscious suggestion.

Hahnemann's first section contains the idea, and he has rightly made it of supreme importance in placing it first. *Health* is first.

"The first and sole duty of the physician is to restore *health* to the sick. This is the true art of healing." He is to form a *true and clear ideal of his mission* and set it constantly before his mind. The physician who permits any other image or ideal to take possession of his imagination is perverting it from its high and holy purpose. How often must it be repeated that disease is not an entity, and the train of evils following this false philosophy met and combatted. Yet physicians will continue to conjure up phantoms of pneumonia, diphtheria, and typhoid fever, with the almost inevitable result of falling into the error of treating the disease instead of the patient. Names assume the importance of things too often, and lead the unwary into error.

The proper use of the imagination will help the physician out of his difficulty.

"I am never confused if I can see far enough," said Emerson. Commenting on this, a recent writer has said, "The imagination is *the faculty which sees.* Of the several faculties by the exercise of which men live it is the most necessary and vital; and yet so little is it understood that it is constantly spoken of as something very beautiful in its activity, but the especial property of artists, poets, and dreamers." Even so, it is the privilege of the homœopathician to be an artist in his work. He will become so by the proper and intelligent cultivation and use of his imagination. Using it properly to form an image of health, strength, and beauty for his patient, and intelligently noting the points in which there is a departure from this true standard, he will be inspired by a desire to make his patient conform. Clear perception of a true standard of health, knowledge of the law of cure, combined with a desire to help, generate courage, hope, faith. In exhibiting and applying these qualities, together with the appropriate remedy, he is wielding the mightiest forces in the universe for good. The very presence of such a physician in the sick-room, by his unconscious influence, gives the patient an impulse toward health, the effects of which will be seen at once. Patients and friends have often observed and spoken of it. He inspires confidence at once. Unconsciously, perhaps, a new idea is set before the mind of the patient, and in his desire to attain it he sets in motion those latent forces of his being which tend to equilibrium and health.

But these faculties may be consciously and intelligently exercised, with greatly enhanced power. The physician so endowed and trained is in a position to instruct his patient how to call into activity these latent qualities and forces, and direct them toward health. Certain patients are said to have recovered through sheer "grit" and will power, who would otherwise have died. Others are said to have "worried themselves to death." It is probably true in both cases. The state and temper of the mind are of the greatest importance. What it shall be often depends upon the patient's knowledge. If he has right views of the nature of disease, and the principles of cure, he will be more at ease. Nothing that the physician knows in this sphere is improper for the patient to know, and it is the physician's mission and duty to teach him. Teach him the Hahnemannian philosophy of these things and how to apply it, and you will not only directly benefit him, but make him your friend and follower.

Imagination must also be used in the study of materia medica, if mastery is to be obtained. The physician who merely reads over symptoms of remedies and cases, and makes a mechanical comparison, will frequently fail in selecting the remedy. Another maxim we frequently hear is that "mere symptom-covering is not enough." We have also heard of certain great ones—masters of the art—who have the rare and precious faculty of "getting at the genius of the remedy." What does it mean? What is the difference between the mere symptom-coverer and the master—the artist who discovers the genius of the remedy? It is simply that one has learned how to use his imagination while studying, and the other has not. The *artist* sits down, reads the proving of a medicine, and studies the symptoms in all their aspects and relations, analyzing, comparing, reflecting, until an image or picture is formed in his imagination which takes form and color and coherency. He *sees* the remedy with the internal eye of his imagination. All its characteristic features stand out before him as he gazes. It becomes real to him. It inspires him. It becomes his familiar friend, whose features he will henceforth recognize wherever seen. It becomes a part of his mental furniture—to be used when called for.

So in examining and studying a case. Again he forms a symptom-picture in his imagination; but his picture takes the form of a remedy, not a "disease." We say, "these symptoms indicate a remedy." Let it be so actually. Let them exist in the mind as a counterpart or reflection of a remedy which must be found. They are the language of the suffering organism, and indicate a loss of harmony, which is accompanied by dis-ease—loss of ease. Nothing more. This will disappear when the cause is removed and harmony restored.

This is not by any means to make light of the patient's sufferings, or to underestimate the gravity of his condition. It takes full cognizance of both, but in such a manner as to act in harmony with the laws of the human mind as well as body.

He who ridicules a patient's sufferings, or tells him they are nothing, or "only imaginary," commits as grave an error as he who treats the disease instead of the patient. His own mind and imagination are dwarfed and distorted. When Hahnemann's injunction to give the mental symptoms the highest rank is remembered, let it not be forgotten that sometimes the mental symptoms of the physician are of quite as much importance as those of the patient.

"Pysician, heal thyself."

